# Evolutionary Analysis of Bile Acid-Conjugating Enzymes Reveals a Complex Duplication and Reciprocal Loss History

**DOI:** 10.1093/gbe/evz238

**Published:** 2019-10-31

**Authors:** Bogdan M Kirilenko, Lee R Hagey, Stephen Barnes, Charles N Falany, Michael Hiller

**Affiliations:** 1 Max Planck Institute of Molecular Cell Biology and Genetics, Dresden, Germany; 2 Max Planck Institute for the Physics of Complex Systems, Dresden, Germany; 3 Center for Systems Biology, Dresden, Germany; 4 Department of Medicine, University of California at San Diego, USA; 5 Department of Pharmacology and Toxicology, Targeted Metabolomics and Proteomics Laboratory, University of Alabama, Birmingham, USA

**Keywords:** gene duplication, reciprocal gene loss, bile acid conjugation

## Abstract

To fulfill their physiological functions, bile acids are conjugated with amino acids. In humans, conjugation is catalyzed by bile acid coenzyme A: amino acid *N*-acyltransferase (BAAT), an enzyme with a highly conserved catalytic triad in its active site. Interestingly, the conjugated amino acids are highly variable among mammals, with some species conjugating bile acids with both glycine and taurine, whereas others conjugate only taurine. The genetic origin of these bile acid conjugation differences is unknown. Here, we tested whether mutations in BAAT’s catalytic triad could explain bile acid conjugation differences. Our comparative analysis of 118 mammals first revealed that the ancestor of placental mammals and marsupials possessed two genes, *BAAT* and *BAATP1*, that arose by a tandem duplication. This duplication was followed by numerous gene losses, including *BAATP1* in humans. Losses of either *BAAT* or *BAATP1* largely happened in a reciprocal fashion, suggesting that a single conjugating enzyme is generally sufficient for mammals. In intact *BAAT* and *BAATP1* genes, we observed multiple changes in the catalytic triad between Cys and Ser residues. Surprisingly, although mutagenesis experiments with the human enzyme have shown that replacing Cys for Ser greatly diminishes the glycine-conjugating ability, across mammals we found that this residue provides little power in predicting the experimentally measured amino acids that are conjugated with bile acids. This suggests that the mechanism of BAAT’s enzymatic function is incompletely understood, despite relying on a classic catalytic triad. More generally, our evolutionary analysis indicates that results of mutagenesis experiments may not easily be extrapolatable to other species.

## Introduction

Bile has been long known to be important in health and disease (Heaton and Morris 1971). It is a watery yellow fluid produced by the liver and excreted into ducts that transport it to the upper small intestine. The dominant components of bile are a family of cholesterol-derived compounds—bile alcohols and bile acids. With a few exceptions, almost all mammals produce bile acids via a pathway that involves multiple enzymes (Russell 2003; Hagey et al. 2010). Bile acids have been shown to have a multiplicity of roles (Hofmann and Hagey 2014). Their secretion from the liver into the bile ducts pulls in water that makes up the bile flow. As a physiological molecule, bile acids are required for the absorption of digested lipids and fat-soluble vitamins in the intestine, which is evident from human patients exhibiting bile acid synthesis defects (Heubi et al. 2007). Furthermore, in the past two decades, there has been a growing appreciation of the role of bile acids as signaling and communication molecules. In particular, bile acids influence the intestinal environment and affect the proportions of the different species of bacteria that make up the gut microbiome of the host (Nie et al. 2015). In turn, the intestinal microbiome alters the structure of bile acids, and when these structures are reabsorbed from the intestine and returned to the bloodstream, they influence host cholesterol, triglyceride, and glucose levels (Schaap et al. 2014), host energy homeostasis (Broeders et al. 2015; DiMarzio et al. 2017), and host immunity (Fiorucci et al. 2018). 

Prior to their secretion into bile, bile acids are conjugated with the amino acids taurine or glycine (Hofmann et al. 2010). Conjugation substantially reduces the passive reabsorption of the molecule through biological membranes. This allows bile acids in the intestine to form micelles, which are necessary for the absorption of lipids (Ekwall 1951; Hofmann 1963). Subsequently, a wide variety of intestinal microbes possess the ability to deconjugate bile acids (Jones et al. 2008), and these deconjugated forms are then returned to the liver for reconjugation.

Bile acid conjugation is catalyzed by the enzyme bile acid coenzyme A: amino acid *N*-acyltransferase (BAAT), which is encoded by the *BAAT* gene. This enzyme has two locations in hepatocytes—peroxisomes (25–30%) where it conjugates newly synthesized bile acids, and the cytosol (70–75%) where it reconjugates bile acids returning from the intestine (Pellicoro et al. 2007; Styles et al. 2007). Bile acid conjugation is a three-step process. To form an amino acid conjugate, initially an acid–anhydride bond is formed between the carboxylic acid of the bile acid and adenosine-5′-monophosphate (Ikegawa et al. 1999). The *O*-adenylate is then exchanged for coenzyme A to form a bile acid-CoA ester. Next, BAAT binds to the bile acid-CoA unit and breaks the CoA linkage, forming a covalent bond between the bile acid and a cysteine in the active site (Sfakianos et al. 2002). In humans, BAAT is the only enzyme capable of bile acid conjugation, because loss-of-function mutations result in familial hypercholanemia, a disease characterized by the absence of bile acid conjugation, growth failure, and vitamin deficiency (Carlton et al. 2003; Setchell et al. 2013).

Critical for the enzymatic function of human BAAT are three amino acids—^235^Cys, ^328^Asp, and ^362^His (Sfakianos et al. 2002). These three amino acids constitute a catalytic triad and have a classic charge-relay system as seen in cysteine- and serine-proteases. Generally, mutations of these amino acids in these enzyme classes abolish activity (Pazirandeh et al. 1991). However, mutagenesis experiments with human BAAT have shown that a change from ^235^Cys to ^235^Ser alters BAAT selectivity for glycine and taurine (Sfakianos et al. 2002). Wild-type human BAAT can utilize either taurine or glycine (Falany et al. 1994), but the ^235^Ser mutant, while retaining its BAAT activity with taurine, has a substantially reduced BAAT activity with glycine as the substrate. Moreover, this mutant also exhibits a marked hydrolase activity against bile acid-CoA substrate (Sfakianos et al. 2002).

Consistent with human BAAT mediating conjugation with either glycine or taurine in vitro (Sfakianos et al. 2002), human bile contains both taurine and glycine conjugates. However, among mammals, the preferred amino acids that are conjugated to bile acids are highly variable (Hagey et al. 2010). Like in humans, several primates (such as great apes, rhesus, and sifaka), several glires (rats, naked mole rat, beaver, and rabbit), and several *Laurasiatheria* (cow, zebra, and pig) utilize either taurine or glycine, although the ratio of glycine to taurine conjugation can vary considerably. In contrast, other primates (such as marmosets and lemurs), other rodents (mouse, chinchilla, and squirrel), other *Laurasiatheria* (cetaceans, goat, carnivora, and bats), and most *Afrotheria* exclusively conjugate bile acids with taurine but not glycine (Hagey et al. 2010). Although the variability in the preferred conjugated amino acids has been well characterized, the genetic origin of this variation is not known.

To address the question of what drives the molecular basis of bile acid conjugation differences among mammals, we reasoned that the results of mutagenesis experiments with human BAAT can be extrapolated to other mammals, because the catalytic triad that is required for enzymatic function is well conserved (Sfakianos et al. 2002). Specifically, we hypothesized that ^235^Cys would be predictive for conjugation with either glycine or taurine, whereas ^235^Ser would be predictive for taurine-only conjugation. By inspecting the active site of BAAT across 118 mammals, we surprisingly found that the *BAAT* gene is inactivated (lost) in many mammals that exhibit bile acid conjugation. A subsequent genomic analysis revealed that *BAAT* exhibits a complex duplication and reciprocal loss history in mammals, and that almost all species preserve at least one intact *BAAT*-like gene. Finally, comparing the active-site residues of all intact *BAAT* and *BAAT*-like genes shows that ^328^Asp and ^362^His are perfectly conserved and that the residue corresponding to position 235 provides little predictive power for observed bile acid conjugation patterns. This suggests that the mechanistic understanding of the enzymatic function of human BAAT cannot easily be extrapolated to other mammals and indicates that additional BAAT amino acid residues affect its selectivity for the amino acid conjugation of bile acids.

## Materials and Methods

### Detecting and Validating the Loss of *BAAT* and *BAATP1* in Mammals

Because *BAAT* exhibits a frameshifting deletion overlapping the active-site residue in *Bovidae*, we analyzed whether the coding sequence of *BAAT* has gene-inactivating mutations across 118 mammals (all species and genome assemblies are listed in supplementary table 1, Supplementary Material online). To this end, we used a previously developed approach that uses genome alignments to detect different types of gene-inactivating mutations, namely premature stop-codon mutations, frameshifting insertions and deletions, splice site disrupting mutations, and large exonic deletions (Sharma, Hecker, et al. 2018). This approach employs a series of filter steps to exclude false inactivation mutations. Briefly, exons that appear deleted or do not align are ignored if the corresponding locus overlaps an assembly gap in the other assembly. Frameshifts that compensate each other and return to the ancestral reading frame are ignored as well. Coding exon-structure aware realigner (CESAR) realignments are used to remove false inactivating mutations caused by alignment ambiguities and to remove false splice site mutations in case of evolutionary splice-site shifts (Sharma et al. 2016, 2017). The same approach was used to analyze inactivating mutations in *BAATP1* orthologs with the following difference. As human *BAATP1* is not an intact gene, we cannot use the human gene structure as a reference. Therefore, we used the Ensembl-annotated cow *BAATP1* (ENSBTAT00000079956.1), which encodes an intact gene, as the reference and used CESAR to realign the three coding exons of cow *BAATP1* to the orthologous loci that aligned to the human *BAATP1* locus. The CESAR alignments were then searched for inactivating mutations. Analysis of relaxed selection (supplementary table 2, Supplementary Material online) was performed using RELAX (Wertheim et al. 2015) and the robust codon alignments of *BAAT* and *BAATP1* genes (below).

**Fig. 1.**
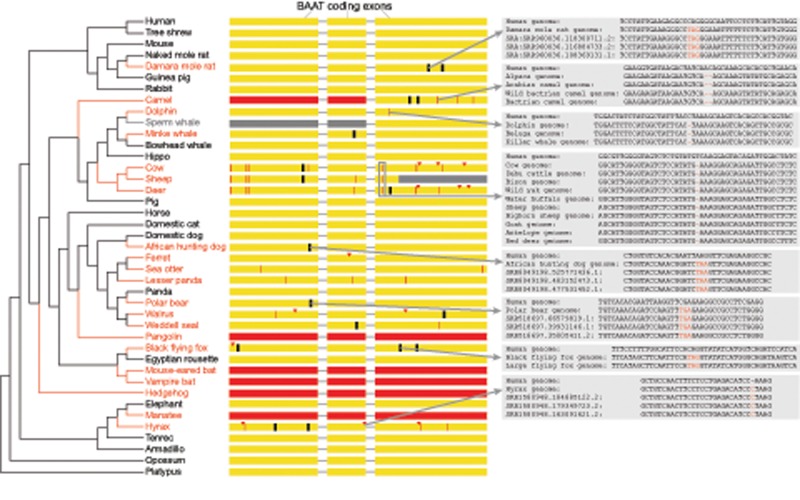
—Repeated losses of *BAAT* in placental mammals. A phylogenetic tree shows the species that have gene-inactivating mutations in *BAAT* (red font) together with a subset of species that have an intact *BAAT* gene (black font). The three coding exons of *BAAT* are shown as yellow boxes together with the position of inactivating mutations. Red arrowheads represent frameshifting insertions, red vertical lines represent frameshifting deletions, black vertical lines represent premature stop codons, and red boxes represent exon deletions. Gray insets illustrate that all shown inactivating mutations are validated either by unassembled sequencing reads or by the presence of the same mutation in the genomes of related species. The downstream parts of exon 3 in sheep (gray color) overlap an assembly gap; however, sheep shares other inactivating mutations with related species, showing that *BAAT* is lost in the sheep. The status of *BAAT* in the sperm whale is uncertain as the genomic region around exons 1 and 2 is not properly assembled (scaffolds end up- and downstream of these exons and there is no colinear alignment). Based on inactivating mutations that are shared between related species, we infer as many as 18 independent losses of *BAAT* have occurred in placental mammals.

**Fig. 2.**
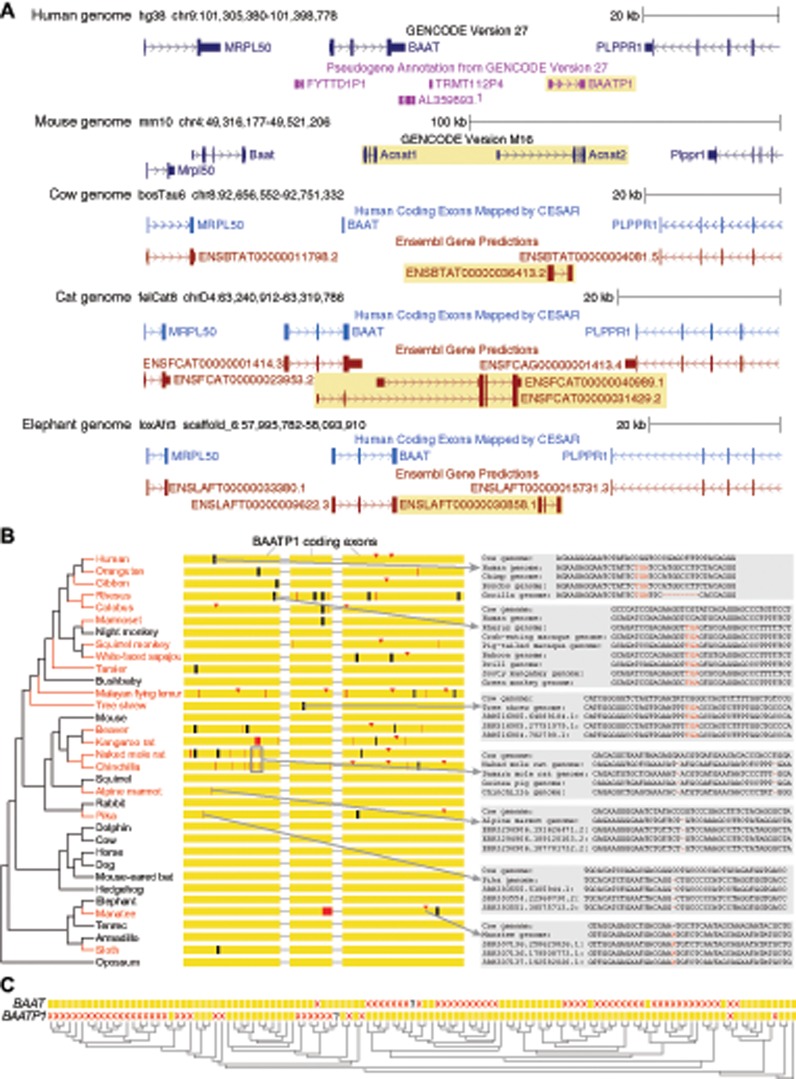
—Evolution of a second *BAAT* gene (*BAATP1*) that arose by a tandem duplication. (*A*) UCSC genome browser (Haeussler et al. 2019) screenshot of the human locus containing *BAAT* and the surrounding genes (*MRPL50* and *PLPPR1*) shows the presence of an intron-containing pseudogene *BAATP1* (yellow background) downstream of *BAAT*, suggesting that this pseudogene copy arose by a tandem duplication. This pseudogene aligns to an intact gene in other placental mammals, as shown by the Gencode or Ensembl gene annotation (Cunningham et al. 2019). In the mouse, a tandem duplication of the human *BAATP1* ortholog occurred, giving rise to two genes annotated as *Acnat1* and *Acnat2*. In addition to Ensembl, we show human genes that are mapped by CESAR to the orthologous cow, cat, and elephant locus via the genome alignment (Sharma et al. 2016; Sharma and Hiller 2017). As CESAR only projects intact human genes, it cannot annotate intact *BAATP1* orthologs (yellow background) in other species. In cow, CESAR only annotates the intact second exon of *BAAT*, as the other exons have inactivating mutations (fig. 1). (*B*) Similar to *BAAT*, *BAATP1* is lost repeatedly in placental mammals (visualization as in fig. 1). (*C*) Visualizing the presence (yellow box) or loss (red cross) of *BAAT* and *BAATP1* in 118 mammals shows that losses of these genes mostly occurred in a reciprocal fashion. A question mark indicates that it is uncertain whether the gene is present or lost.

As putative gene-inactivating mutations can be base errors in genome assemblies, we validated inactivating mutations as follows. Inactivating mutations that are identical between at least two independently sequenced and assembled sister species (see figs. 1 and 2) are most likely real mutations that already occurred in the common ancestor. For inactivating mutations that occur only a single genome assembly, we used unassembled sequencing reads from the NCBI TRACE and Sequence Read Archives (Kodama et al. 2012) for validation. To this end, we extracted the genomic context up- and downstream of the inactivating mutation and used megablast (parameters match score 1, mismatch scores −2, gap costs linear, expectation value threshold 10) to determine the number of reads that support this mutation, as done before (Hecker et al. 2017, 2019; Sharma, Lehmann, et al. 2018). Putative mutations that are not supported by reads are most likely base errors in the assembly, as illustrated in supplementary figure 1, Supplementary Material online. SRA accessions are provided in supplementary table 1, Supplementary Material online.

### Analyzing *BAAT* Genes in Platypus and Nonmammalian Amniotes

We aligned the platypus VGP assembly (Zhang et al. 2019) to the human hg38 genome by applying lastz (Harris 2007) with parameters *K* = 2,400, *L* = 3,000 and the HoxD55 scoring matrix, axtChain (Kent et al. 2003), and chainCleaner (Suarez et al. 2017) (both with default parameters). Collinear alignment chains were visualized in the UCSC genome browser (Casper et al. 2018). Using this genome alignment, we projected human genes to the platypus with CESAR (Sharma et al. 2016, 2017). In addition, we visualized the platypus NCBI gene annotation (Sayers et al. 2019) (downloaded from NCBI) in the UCSC genome browser. This analysis showed gene order rearrangements and only a single *BAAT* gene.

**Fig. 3.**
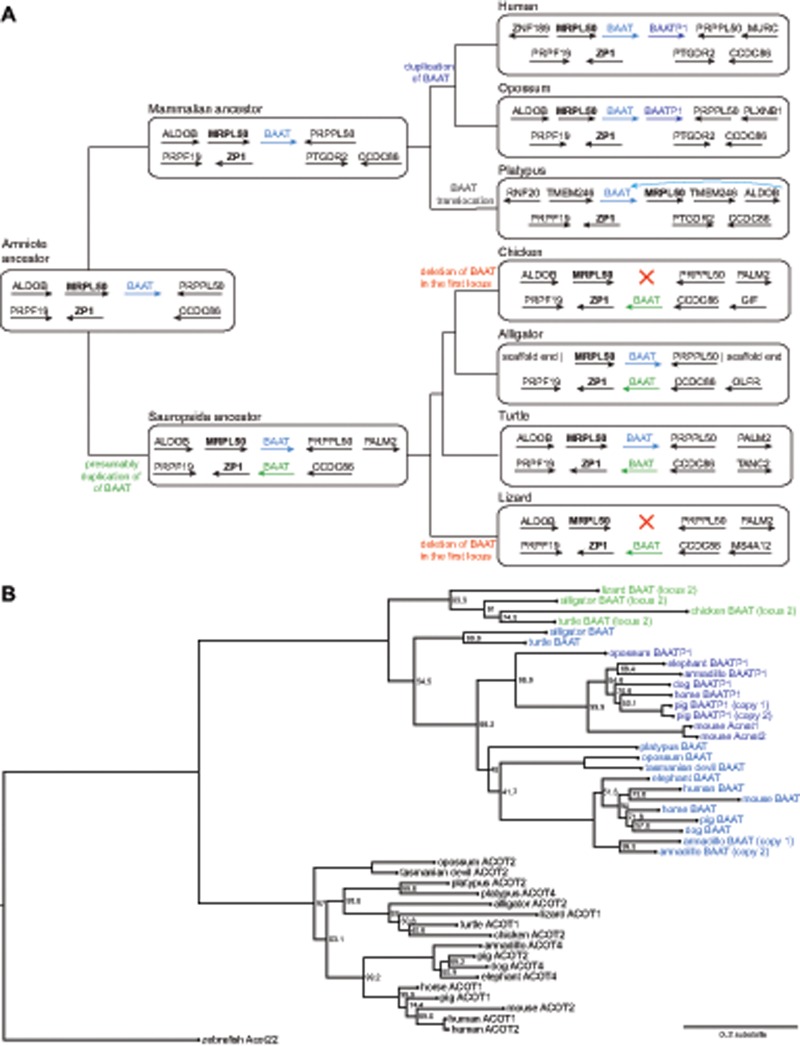
—Duplication and reciprocal loss patterns of *BAAT* during amniote evolution. (*A*) Simplified phylogenetic tree of representative amniote species where we analyzed gene order conservation of both loci containing annotated *BAAT* genes. The gene order of ancestral lineages was inferred by parsimony and major gene loss or rearrangement events are labeled on the respective branches. The amniote ancestor likely possessed a single *BAAT* gene (blue font), which was flanked by *MRPL50* (bold font). A tandem duplication of this gene occurred before the split of placental mammals and marsupials, giving rise to *BAAT* and *BAATP1*. Turtle and alligator exhibit a second *BAAT* gene (green font) in a genomic locus flanked by *ZP1* (bold font). As frog and fish do not appear to possess a *BAAT*-like gene in this locus, this second *BAAT* gene likely arose by duplication in the *Sauropsida* ancestor. The *BAAT* gene in the first locus was likely independently lost in the lineage leading to chicken and anole lizard. In platypus, gene order around the single *BAAT* gene differs from other lineages, suggesting rearrangements in this locus that involved a local translocation of *BAAT* (note that the *MRPL50* gene is downstream of *BAAT*). (*B*) Phylogenetic tree computed by phyML (Yang 2007) from *BAAT* and *ACOT* type 1 family members corroborates the duplication and loss history of *BAAT* genes inferred gene order analysis. Bootstrap values in percent of 1,000 iterations are shown on those branches where bootstrap support was <100%. Zebrafish *Acot22* was used as an outgroup to root the tree. The tree supports that *BAAT* genes are separated from other *ACOT* family members and that the *BAAT* in both loci (blue and green, see panel *A*) represent two groups. *BAATP1* genes of placental mammals and marsupials (dark blue font) form a well-supported group, supporting that a single tandem duplication occurred. Several placental mammals exhibit lineage-specific duplications of *BAAT* (armadillo) or *BAATP1* (mouse, pig).

To understand the evolutionary history of *BAAT* in amniotes, we analyzed the genomic context of this gene in representative amniotes: American alligator (allMis1 assembly), chicken (galGal4), Anole lizard (anoCar2), and painted turtle (chrPic2). Chains of colinear local alignments (Kent et al. 2003) between hg38 and these assemblies were previously computed with highly sensitive alignment parameters (Sharma and Hiller 2017) and were used to locate genomic loci in these species that is orthologous to the human *BAAT*/*BAATP1* locus. For these genomic loci, we obtained the order and orientation of annotated genes, using the Ensembl gene annotation (Cunningham et al. 2019) and human genes projected via CESAR (Sharma et al. 2016, 2017). As the chicken and lizard locus that is orthologous to the human *BAAT*/*BAATP1* locus did not contain a *BAAT* gene (this is confirmed by the latest chicken galGal6 assembly), we queried the Ensembl database, which revealed a gene annotated as a 1:1 ortholog to human *BAAT* (chicken ENSGALG00000040619, lizard ENSACAG00000017812). This gene is contained in the second locus, flanked by *ZP1* (fig. 3). As both alligator and turtle also have a *BAAT* gene in this locus, this suggests that the amniote ancestor possessed two *BAAT* genes in two different loci and that chicken and lizard have independently lost the gene located in the first locus.

### Inferring a Tree of *BAAT* and *ACOT* Genes

To corroborate the duplication/loss history of *BAAT* genes that we inferred from the analysis of conserved gene order and to provide additional evidence that *BAAT* gene in the second locus is not a different member of the larger *ACOT* type 1 gene family, we build a gene tree. To this end, we aligned the sequences of different *ACOT* and *BAAT* genes from placental mammals, opossum, platypus, chicken, alligator, turtle, and lizard (supplementary tables 3 and 4, Supplementary Material online). The zebrafish *Acot22* sequence was used as an outgroup to root the tree. The sequences were aligned with MAFFT (Katoh and Standley 2013) (default parameters), and poorly aligning regions were removed using TrimAL (Capella-Gutierrez et al. 2009) (parameter -nogaps). Then, we generated a gene tree using phyML (Yang 2007) with 1,000 bootstrap replicates. We used Notung 2.9 (Darby et al. 2017) to reconcile the gene tree with the species tree.

### Reconstructing Ancestral Amino Acids in the Active Center

**Fig. 4.**
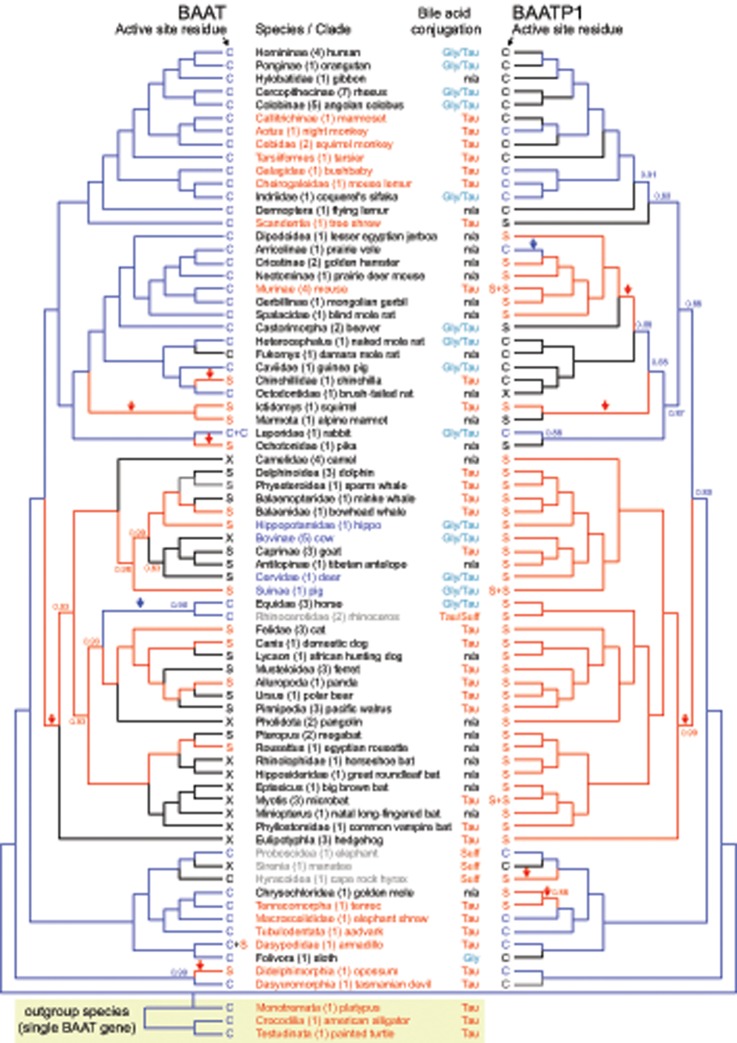
—Active-site residue in mammalian BAAT and BAATP1. The active-site residue corresponding to position 235 in human BAAT is indicated as S (Ser, red font) or C (Cys, blue font). For species that have inactivated *BAAT* or *BAATP1* genes, the active-site residue is shown in black font, with X representing a deletion of the residue at position 235. For the *Murinae*, rabbit, pig, *Myotis* bats and armadillo, two residues are shown, because these species exhibit a lineage-specific duplication of *BAAT* or *BAATP1*. The color of branches in the gene trees indicates the reconstructed ancestral amino acid using the same color code (Ser, red; Cys, blue; black, lost gene). The posterior probability of the reconstructed ancestral amino acid is indicated for all branches where this probability is <0.99. Arrows highlight the branches along which a mutation from the ancestral Cys to Ser (red arrow) or a mutation from Ser to Cys (blue arrow) was inferred. The bile acid conjugation profile is taken from Hagey et al. (2010). Lineages that contradict the prediction that Cys-containing *BAAT* or *BAATP1* gene confers the ability to conjugate either glycine or taurine are shown in red font. Lineages that contradict the prediction that the presence of Ser-containing *BAAT*/*BAATP1* gene(s) confers only ability to conjugate taurine are shown in blue font. Rhinoceroses, elephants, manatees, and rock hyraxes are shown in gray font because these species are special, in that they do not produce C_24_ bile acids but only C_27_ bile alcohols that are conjugated to sulfate (Sulf) or taurine.

To reconstruct the ancestral amino acids in the active center, we build two multiple codon alignments, using the *BAAT* or *BAATP1* genes of placental mammals and marsupials. We added the single *BAAT* gene of platypus, alligator, and turtle as outgroup sequences to both alignments. As the accuracy of ancestral reconstruction increases with the number of species, we also included the *BAAT* or *BAATP1* sequences of species that lost these genes. To this end, we masked all in-frame stop codons and codons overlapping frameshifting insertions or deletions by replacing them with NNN in the respective species. In addition, we also masked the two codons flanking a frameshift because the position of the frameshifting insertion and deletion is sometimes ambiguous. To increase the alignment robustness, we further aligned the sequences with three aligners (PRANK [Loytynoja 2014], MUSCLE [Edgar 2004], and MAFFT [Katoh and Standley 2013]) and masked all codons in all species that are not consistently aligned by all three methods. These robust codon alignments of *BAAT* and *BAATP1* genes are provided in supplementary tables 5 and 6, Supplementary Material online. To reconstruct ancestral amino acids, we applied codeml (Yang 2007) to these alignments. The reconstructed ancestral states of position 235 are shown in figure 4 together with the probabilities of each amino acid.

### Searching Other ACOT Type-1 Family Members

For hippopotamus, cow, deer, and pig, we searched for other ACOT type-1 family members that have a cysteine in the active center. To this end, we used NCBI protein BLAST (Max target sequences = 1,000, word size = 3) to search for homologs to the human BAAT protein sequence. No ACOT type-1 protein with a cysteine-based active center was found. This analysis was corroborated by inspecting all local alignment chains that align to the third *BAAT* exon in human.

## Results

### Widespread Losses of *BAAT* in Mammals

To find the genetic basis for the variation in the conjugated amino acids, we inspected the active-site residue at position 235 in *BAAT* of 118 mammals. Surprisingly, we noticed that this residue overlaps a frameshifting 1-bp deletion in *Bovidae*, suggesting that *BAAT* might be inactivated in this lineage. Therefore, we first analyzed in which of the 118 mammals is this gene actually conserved (supplementary table 1, Supplementary Material online). This analysis revealed that *BAAT* has undergone gene-inactivating mutations, such as frameshifting insertions and deletions, premature stop codons, splice-site mutations, and exon deletions in the genomes of at least 45 mammals (fig. 1).

Being aware that sequencing and assembly errors can mimic gene loss (Hecker et al. 2017, 2019; Sharma, Lehmann, et al. 2018), we manually validated the putative loss of *BAAT* in every species as follows. First, we validated the correctness of all inactivating mutations to rule out sequencing errors that can be present in genome assemblies. We found that in many cases, identical inactivating mutations are shared between independently sequenced and assembled genomes of related species (insets in fig. 1), which supports the validity of these mutations. Furthermore, we used unassembled sequencing reads to confirm all inactivating mutations for assemblies where no closely related sister species genomes are available. With exception of the pig, where the only inactivating mutation turned out to be a base error (supplementary fig. 1, Supplementary Material online), we could confirm all other inactivating mutations in the other 44 mammals (supplementary table 1, Supplementary Material online). Second, for all 44 mammals, the remnants of the inactivated *BAAT* gene occur in the context of conserved gene order. Together, validated inactivating mutations and conserved gene order suggest that *BAAT* has been lost in 44 placental mammals. Based on the presence of shared inactivating mutations, we infer that *BAAT* may have been inactivated as many as 18 times in the branches of mammalian evolution (fig. 1).

### A Second *BAAT*-Like Gene Explains Bile Acid Conjugation in *BAAT*-Loss Species

These widespread losses of *BAAT* are unexpected because almost all *BAAT*-loss species exhibit conjugated bile acids (Hagey et al. 2010) and in humans, *BAAT* is the only gene capable of bile acid conjugation (Carlton et al. 2003; Setchell et al. 2013). Therefore, we investigated which gene could be responsible for bile acid conjugation in *BAAT*-loss species. Inspection of the larger locus around *BAAT* in humans revealed the presence of an intron-containing pseudogene annotated as *BAATP1* (BAAT pseudogene 1, fig. 2*A*) that contains several inactivating mutations in humans. Interestingly, this human pseudogene locus aligns to a genomic region in other placental mammals that contains a second intact *BAAT*-like gene, as shown in figure 2*A* for mouse, cow, cat, and elephant. This suggests that a tandem duplication of an ancestral *BAAT*-like gene occurred before the split of these placental mammals, giving rise to *BAAT* and *BAATP1*, which was followed by the loss of *BAATP1* in humans. In the mouse, another tandem duplication of *BAATP1* happened, giving rise to two genes annotated as *Acnat1* and *Acnat2*. Although *Acnat2* has not been experimentally studied, previous experiments showed that mouse *Acnat1* is able to conjugate bile acids or fatty-acid CoA to taurine, even though its activity is much lower for bile acids (Reilly et al. 2007). This shows that intact orthologs of human *BAATP1* can function as a second bile acid-conjugating enzyme. Consistent with this observation, previous biochemical experiments have shown that the cow possesses a functional enzyme with *N*-acyltransferase activity, although the identity of the protein was not determined (Vessey 1979). Our analysis suggests that the intact *BAATP1* ortholog in cow (Ensembl ENSBTAG00000025760) most likely encodes the bile acid-conjugating enzyme, providing an explanation for why conjugated bile acids were observed in many mammals that have lost *BAAT*. In a similar manner, the loss of *BAATP1* in humans explains why *BAAT* is their only gene capable of bile acid conjugation. To indicate that both genes arose by a tandem duplication of an ancestral *BAAT* gene, we consistently refer to both genes as *BAAT* and *BAATP1*, despite the fact that *BAATP1* is an intact gene in many mammals.

### Widespread Reciprocal Losses of *BAATP1* in Placental Mammals

Because *BAATP1* is an inactivated gene in humans, we analyzed in detail in which mammals this gene is also lost. Putative inactivating mutations were validated by sequencing reads and the presence of the same mutation in related species, as described above. This analysis showed that *BAATP1* is lost not only in humans but in total eight times independently in the primate lineage (fig. 2*B*). Furthermore, *BAATP1* is lost in many other placental mammal lineages, comprising a total of 34 mammals in our data set. Based on inactivating mutations that are shared between related species, we estimate that 17 independent losses of *BAATP1* occurred in placental mammals.

Next, we tested whether the remaining sequences of inactivated *BAAT* or *BAATP1* genes evolve under relaxed selection. Using RELAX (Wertheim et al. 2015), we found significant evidence of relaxed selection for one of 13 *BAAT*-loss lineages and seven of 17 *BAATP1*-loss lineages (supplementary table 2, Supplementary Material online). Several factors could explain the absence of significant evidence for relaxed selection in gene-loss lineages. First, for recently inactivated genes, the power to detect significant evidence for a shift from purifying selection to relaxed selection or neutral evolution is limited. Indeed, several mammalian lineages may have lost *BAAT* or *BAATP1* more recently. For example, *BAAT* in *Delphinoidea* and the Minke whale exhibits only a single inactivating mutation; however, available RNA-seq data of liver tissue show that the inactivated *BAAT* is not expressed anymore (supplementary fig. 2, Supplementary Material online), supporting the loss of the gene. Additionally, significant evidence for relaxation may be harder to detect for slowly evolving lineages. This is exemplified by *BAAT* in the *Delphinoidea* lineage, where RELAX estimates that 82% of the gene evolves neutrally (Ka/Ks = 1) but evidence for relaxation is not significant (*P*-value 0.08). Second, the detection of relaxed selection in a gene-loss lineage relies on a comparison to all species that have an intact *BAAT*/*BAATP1*, making the assumption that the gene evolves under purifying selection in all species with intact gene(s). If, however, either *BAAT* or *BAATP1* alone would be sufficient for bile acid conjugation (see next paragraph), then this assumption might not hold for some of the species that possess two intact genes. Cat may be such an example. The cat possesses intact *BAAT* and *BAATP1* genes, but available liver RNA-seq data show that *BAAT* is not expressed anymore (supplementary fig. 3, Supplementary Material online), suggesting that *BAATP1* is the major bile acid-conjugating enzyme.

Interestingly, losses of *BAAT* and *BAATP1* are largely reciprocal, with 74 species having lost either *BAAT* or *BAATP1* (fig. 2*C*), which supports one gene is generally sufficient for bile acid conjugation. Although the term “reciprocal gene loss,” defined as “the situation when two lineages that have inherited a gene duplication independently lose alternative members of the duplicated pair after speciation” (Semon and Wolfe 2007), was originally used to describe patterns of gene losses after a whole-genome duplication (Scannell et al. 2006; Semon and Wolfe 2007), our analysis reveals an example where reciprocal losses occurred following a tandem gene duplication. Only two species have lost both genes, the manatee and the Damara mole rat. For the manatee, previous measurements have shown that this species does not produce bile acids at all (similar to the related elephant and rock hyrax), but instead produces bile alcohols that are conjugated with sulfate (Kuroki et al. 1988). This suggests that both *BAAT* and *BAATP1* became obsolete in manatee, which led to the loss of both genes. For the Damara mole rat, the bile composition has not yet been characterized, making this species an attractive target to investigate whether it produces bile acids and if so whether they are conjugated. Apart from the double loss of both genes in these animals, the widespread reciprocal losses of *BAAT* and *BAATP1* suggest that almost all placental mammal lineages preserve at least one intact *BAAT*-like gene, which is consistent with observations that bile acid conjugation occurs in almost all mammals (Hagey et al. 2010).

### Complex Duplication and Loss History of *BAAT* Genes in Amniotes

Next, we asked when the tandem duplication that gave rise to *BAAT* and *BAATP1* occurred. To this end, we analyzed this locus in the genomes of two marsupials (opossum and Tasmanian devil [Mikkelsen et al. 2007; Murchison et al. 2012]) and one monotreme (platypus). We found that opossum possesses intact *BAAT* and *BAATP1* genes in this locus (fig. 3*A*). Likewise, the Tasmanian devil possesses *BAAT* and *BAATP1*, even though *BAATP1* exon 1 is not present in the genome due to an assembly gap. As the previous Sanger sequencing-based platypus assembly (Warren et al. 2008) contained the *BAAT*/*BAATP1* locus on several scaffolds, which prevented a conclusive analysis, we analyzed a new reference-quality platypus assembly produced by the Vertebrate Genome Project (Zhang et al. 2019) where the entire locus is contained on an ∼9.3-Mb contig. Analyzing this assembly revealed several gene order rearrangements but only a single *BAAT* gene in the platypus (supplementary fig. 4, Supplementary Material online). This suggests that the tandem duplication that gave rise to *BAAT* and *BAATP1* occurred after the split of monotremes from the ancestor of both marsupials and placental mammals (fig. 3*A*). This finding is corroborated by an analysis of nonmammal genomes (alligator and turtle), which revealed a single *BAAT* gene in this locus in both species.

Interestingly, in contrast to alligator and turtle, two other nonmammals (chicken and green anole lizard) do not possess any *BAAT* gene in this locus. However, both chicken and lizard have an annotated *BAAT* gene in a second locus (fig. 3*A*). To investigate the evolutionary history of *BAAT* in amniotes, we analyzed gene order in both of these loci. As summarized in figure 3*A*, this analysis infers that the *Sauropsida* ancestor possessed two *BAAT* genes in two distinct genomic loci that are flanked by *MRPL50* in the first locus and by *ZP1* in the second locus. Both alligator and turtle maintain a *BAAT* gene at both loci. Thus, the most likely scenario explaining the absence of *BAAT* in the first locus in chicken and lizard is an independent loss in the lineages leading to the chicken and anole lizard. These findings are confirmed by reconstructing a gene tree, which supports that the *BAAT* genes at both loci are distinct from each other (fig. 3*B*). Importantly, both gene tree and phylogenetic reconciliation support that the *BAAT*/*BAATP1* genes of placental mammals and marsupials arose by a single tandem duplication (fig. 3*B* and supplementary fig. 5, Supplementary Material online). Overall, these results suggest that *BAAT* genes exhibit a complex duplication and loss history not only in placental mammals but also during the evolution of amniotes.

### High Variability of Cys and Ser in the Active Site in Mammalian *BAAT* and *BAATP1*

Having established that *BAAT* and *BAATP1* are two genes likely capable of bile acid conjugation in placental mammals and marsupials, we investigated the active-site residues in all intact *BAAT* and *BAATP1* genes. We found that ^328^Asp and ^362^His are perfectly conserved in intact *BAAT* and *BAATP1* genes. Focusing on position 235, we found that all intact *BAAT* and *BAATP1* orthologs have either Cys or Ser in the active site (fig. 4), which is consistent with either Cys or Ser being necessary for enzymatic activity (Pazirandeh et al. 1991; Sfakianos et al. 2002). However, we observed many changes between Cys and Ser at this position among intact *BAAT* and *BAATP1* orthologs. Using a codon alignment of *BAAT* or *BAATP1*, we reconstructed ancestral sequences, which showed that ^235^Cys is the amino acid that was likely present in the ancestor of placental mammals and marsupials for both BAAT and BAATP1 (fig. 4). This is consistent with the single *BAAT* gene of platypus, alligator, and turtle, which also exhibit ^235^Cys. From the reconstructed ancestral states, we infer that *BAAT*^235^Cys was mutated to Ser in five mammalian lineages and *BAATP1* Cys was mutated to Ser in five mammalian lineages (fig. 4). Back mutations from Ser to Cys happened only once in *BAAT* (in the ancestor of horses and the rhinoceros) and once in *BAATP1* (in the prairie vole).

### Cys or Ser in the Active Site Has Little Power to Predict the Preferred Conjugated Amino Acids

Next, we tested whether the presence of ^235^Cys is predictive for conjugation with either glycine or taurine and whether the presence of ^235^Ser is predictive for taurine-only conjugation. The composition of bile acids and the conjugated amino acids have been experimentally determined for a total of 79 mammals in our data set or their close relatives (Hagey et al. 2010). We intersected these data with the active-site residue of intact *BAAT*/*BAATP1* genes (fig. 4 and supplementary table 1, Supplementary Material online). This revealed several clades or species (Old World monkeys, sifaka, beaver, naked mole rat, rabbit, and horse) possess at least one Cys-containing *BAAT*/*BAATP1* gene and, as predicted, exhibit conjugation with either glycine or taurine. Similarly, several other clades and species (chinchilla, squirrel, *Cetacea*, goats, *Carnivora*, *Chiroptera*, and *Eulipotyphla*) possess only Ser-containing *BAAT*/*BAATP1* gene(s) and, as predicted, exhibit taurine-only conjugation.

However, there are numerous exceptions where the observed conjugation pattern differed from conjugation pattern predicted from the presence of Cys- or Ser-containing BAAT/BAATP1 enzymes. For example, several primates (marmoset, tarsier, bushbaby, and mouse lemur), the tree shrew, mouse, several *Afrotheria* (tenrec, elephant shrew, and aardvark), armadillo, and the two analyzed marsupials (opossum and Tasmanian devil) all possess at least one Cys-containing *BAAT*/*BAATP1* gene, which should confer the ability to conjugate both glycine and taurine, but exhibit taurine-only conjugation (these species are shown in fig. 4 in red font). Furthermore, the *BAAT* gene of the platypus, alligator, and turtle also has a Cys, but these species only conjugate taurine to bile acids.

Even more strikingly, we also observed several violations to the prediction that Ser-containing BAAT/BAATP1 enzymes are only able to conjugate taurine. Hippopotamus, *Bovinae*, deer, and pig possess only Ser-containing BAAT or BAATP1 but exhibit measurable bile acid conjugation with both taurine and glycine (fig. 4, species in blue font). Overall, this analysis shows that residue corresponding to position 235 provides little predictive power for observed bile acid conjugation patterns in mammals and nonmammal species.

## Discussion

In this study, we investigated the evolution of the bile acid amino acid-conjugating *BAAT* gene in 118 mammals to explain their conjugation differences. Unexpectedly, we found that *BAAT* exhibits a complex evolutionary history, characterized by a tandem duplication before the split of placental mammals and marsupials giving rise to *BAAT* and *BAATP1*, which was followed by many independent and mostly reciprocal losses. Tandem duplications of *BAAT* or *BAATP1* subsequently happened in five individual placental mammal lineages (*Murinae*, rabbit, pig, *Myotis* bats, and armadillo), which suggests that this locus is generally prone to tandem duplications. Numerous reciprocal losses of *BAAT* or *BAATP1* in placental mammals suggest that an additional gene copy does not provide an advantage for most species, and that a single gene is generally sufficient for bile acid conjugation. We found only two species (manatee and Damara mole rat) that have lost both *BAAT* and *BAATP1*. Although manatee is special in that it does not produce bile acids but instead sulfate-conjugated bile alcohols (Kuroki et al. 1988), the bile of the Damara mole rat has not been characterized, making this species an attractive target to determine whether it produces bile acids and, if so, whether they are unconjugated.

The mechanism underlying the enzymatic function BAAT appears to be well understood, because the three amino acids in the active site (^235^Cys/Ser, ^328^Asp, and ^362^His) form a catalytic triad (Sfakianos et al. 2002), a very common and highly conserved configuration found at the active site of hydrolase and transferase enzymes (Dodson and Wlodawer 1998; Buller and Townsend 2013). Furthermore, mutagenesis experiments with human BAAT have shown that replacing ^235^Cys with Ser diminishes its ability to conjugate with glycine. This is consistent with the Cys-containing BAAT of the rat being able to conjugate both glycine and taurine (He et al. 2003), whereas the Ser-containing mouse Acnat1 (BAATP1) enzyme is only able to conjugate taurine (Reilly et al. 2007). Given this apparently well-established paradigm, a surprising result of our large-scale comparative study is that the presence of Cys or Ser in BAAT/BAATP1 has little power in predicting the preferred amino acids that are conjugated to bile acids.

Specifically, our analysis revealed a number of mammalian lineages that possess Cys-containing *BAAT* or *BAATP1* genes but conjugate only with taurine. Despite the predicted ability to conjugate either glycine or taurine, it is possible that all these species only conjugate with taurine, because glycine is not readily available in the peroxisomes of hepatocytes. Indeed, the availability of taurine and glycine influences the ratio of taurine and glycine conjugates (Sweeny et al. 1991). However, we do not favor this hypothesis because in vitro experiments with mouse BAAT have shown that the mouse enzyme is only able to conjugate taurine even if glycine is available (Falany et al. 1997).

Maybe even more surprising, we found that the hippopotamus, *Bovinae*, deer, and pig exhibit conjugation with either glycine or taurine, despite lacking any Cys-containing *BAAT* or *BAATP1* gene. Several hypotheses could explain this observation. First, it is possible that a third enzyme is capable of conjugating bile acids to glycine. However, we believe that this is less likely, because in humans, which naturally lost *BAATP1*, BAAT is the only enzyme capable of bile acid conjugation (Carlton et al. 2003; Setchell et al. 2013). Furthermore, we searched for other ACOT type 1 family members with a cysteine in the active site and found none in these lineages. A second hypothesis is that BAAT or BAATP1 of these lineages is able to conjugate glycine with a serine-based mechanism. For example, a previous study showed that the C-terminus of the enzyme also influences the ratio between taurine and glycine conjugation (Styles et al. 2016); however, we found no obvious correlation between the C-terminal amino acids and the conjugation profile (supplementary table 1, Supplementary Material online). Furthermore, another mechanism not relying on the catalytic triad may exist. For example, the related ACOT type 2 enzymes rely on a “hot-dog” fold for enzymatic activity (Cantu et al. 2014). A third hypothesis relates to the fact that hippopotamus, *Bovinae*, and deer are obligate herbivores. The plant-based diet provides these herbivores with larger amounts of oxalate, which can be harmful as mammals cannot further metabolize oxalate. To compensate for the high intake of oxalate, herbivores prevent endogenous oxalate production by transaminating the oxalate precursor, glyoxylate, to glycine (Danpure 1997). This reaction is mediated by the enzyme AGT (alanine-glyoxylate aminotransferase). As glyoxylate is mainly produced in peroxisomes, many herbivores retarget AGT from mitochondria to peroxisomes (Danpure 1997). Because the peroxisome is the site of both glyoxylate synthesis and bile acid conjugation, the local concentration of glycine in the peroxisome could be very high in herbivores and this glycine would be readily available for BAAT. As human Ser-mutated BAAT exhibits low levels of glycine conjugation (see figure 5 of Sfakianos et al. [2002]), high glycine concentrations may result in bile acids with measurable glycine conjugation. This hypothesis could be tested by in vitro characterizations of cow *BAATP1* under conditions of high glycine concentrations.

Overall, our results suggest that the mechanistic understanding of the enzymatic function of mammalian BAAT may be incompletely understood. More generally, our analysis indicates that results of mutagenesis experiments obtained from enzymes of selected mammals (such as human or mouse) cannot always be extrapolated to other mammals, even if enzymatic function involves a highly conserved active-site configuration such as the catalytic triad.

Finally, an interesting question is why most mammals prefer taurine-only conjugation. One factor might be related to the fact that glycine-conjugated bile acids start to precipitate below a pH of 3, whereas taurine conjugates remain soluble up to a pH of 1.5 (Fini and Roda 1987; Mukaisho et al. 2014). As the acidity of the stomach differs among species, for example, herbivores tend to have higher pH values than carnivores (Beasley et al. 2015), taurine conjugates might be preferred in species exhibiting a more acidic gastrointestinal environment. Another intriguing hypothesis is that taurine (in contrast to glycine) contains sulfur. As bile acids are deconjugated by the gut microbiome, taurine-conjugated bile acids deliver a sulfur-containing compound to the intestinal environment. Gut bacteria can use sulfur-containing compounds, such as taurine, as electron sinks in respiratory metabolism (Laue et al. 1997). Therefore, it is possible that taurine conjugation is beneficial for the host by indirectly providing energy to the gut microbiome, which in turn may contribute to a healthy gut microbiome composition.

## Supplementary Material

Supplementary data are available at *Genome Biology and Evolution* online.

## Supplementary Material

evz238_Supplementary_Data
