# Supplementary material for: Evolutionary Analysis of Bile Acid-Conjugating Enzymes Reveals a Complex Duplication and Reciprocal Loss History
Source: Genome Biol Evol. 2019 Oct 31;11(11):3256–68. doi: 10.1093/gbe/evz238 (PMC6934887; doi:10.1093/gbe/evz238)
Supplement: evz238_Supplementary_Data [file evz238_supplementary_data.zip › Supplement.pdf]

Supplementary Information for

Evolutionary analysis of bile acid-conjugating enzymes reveals a complex duplication and reciprocal loss history

Bogdan Kirilenko [1,2,3], Lee R Hagey [4], Stephen Barnes [5], Charles N. Falany [5], Michael Hiller [1,2,3*]

[1]Max Planck Institute of Molecular Cell Biology and Genetics, Dresden, Germany
[2]Max Planck Institute for the Physics of Complex Systems, Dresden, Germany
[3]Center for Systems Biology Dresden, Germany
[4]University of California San Diego, USA
[5]University of Alabama at Birmingham, USA

Correspondence should be addressed to MH: hiller@mpi-cbg.de

The Supplementary Information contains
- Figures 1-5

Supplementary Tables 1-6 are provided as sheets in a separate Excel file.

Human (hg38) genome	CCTATTGAAGAGGCCCAGGGGCAATTCCTCTTCATTGTAGGAGAAGGTGATAAG
Pig (susScr11) genome	CCCATTGAAAAGGCCCAGGGACATTTCCT-TTCATTGTGGGAGAAGAAGATAAG
Pig (susScr3) genome	CCCATTGAAAAGGCCCAGGGACATTTCCTTTTCATTGTGGGAGAAGAAGATAAG
SRA:SRR652354.17099671.1	CCCATTGAAAAGGCCCAGGGACATTTCCTTTTCATTGTGGGAGAAGAAGATAAG
SRA:SRR652354.20448939.2	CCCATTGAAAAGGCCCAGGGACATTTCCTTTTCATTGTGGGAGAA
SRA:SRR652353.28180105.2	CCCATTGAAAAGGCCCAGGGACATTTCCTTTTCATTGTGGGAGAAGAAGATAAG
SRA:SRR652353.6622181.2	CCCATTGAAAAGGCCCAGGGACATTTCCTTTTCATTGTGGGAGAAGAAGATAAG
SRA:SRR652353.1497213.1	CCCATTGAAAAGGCCCAGGGACATTTCCTTTTCATTGTGGGAGAAGAAGATAAG

Supplementary Figure 1: Base error in the pig susScr11 genome assembly.

The 1 bp frameshifting deletion in *BAAT* exon 3 in the pig susScr11 assembly (red font) is not present in the previous susScr3 assembly. Furthermore, unassembled sequencing reads from the Short Read Archive (SRX219275, SRX219274) do not support this mutation, showing that it is a base error in susScr11.

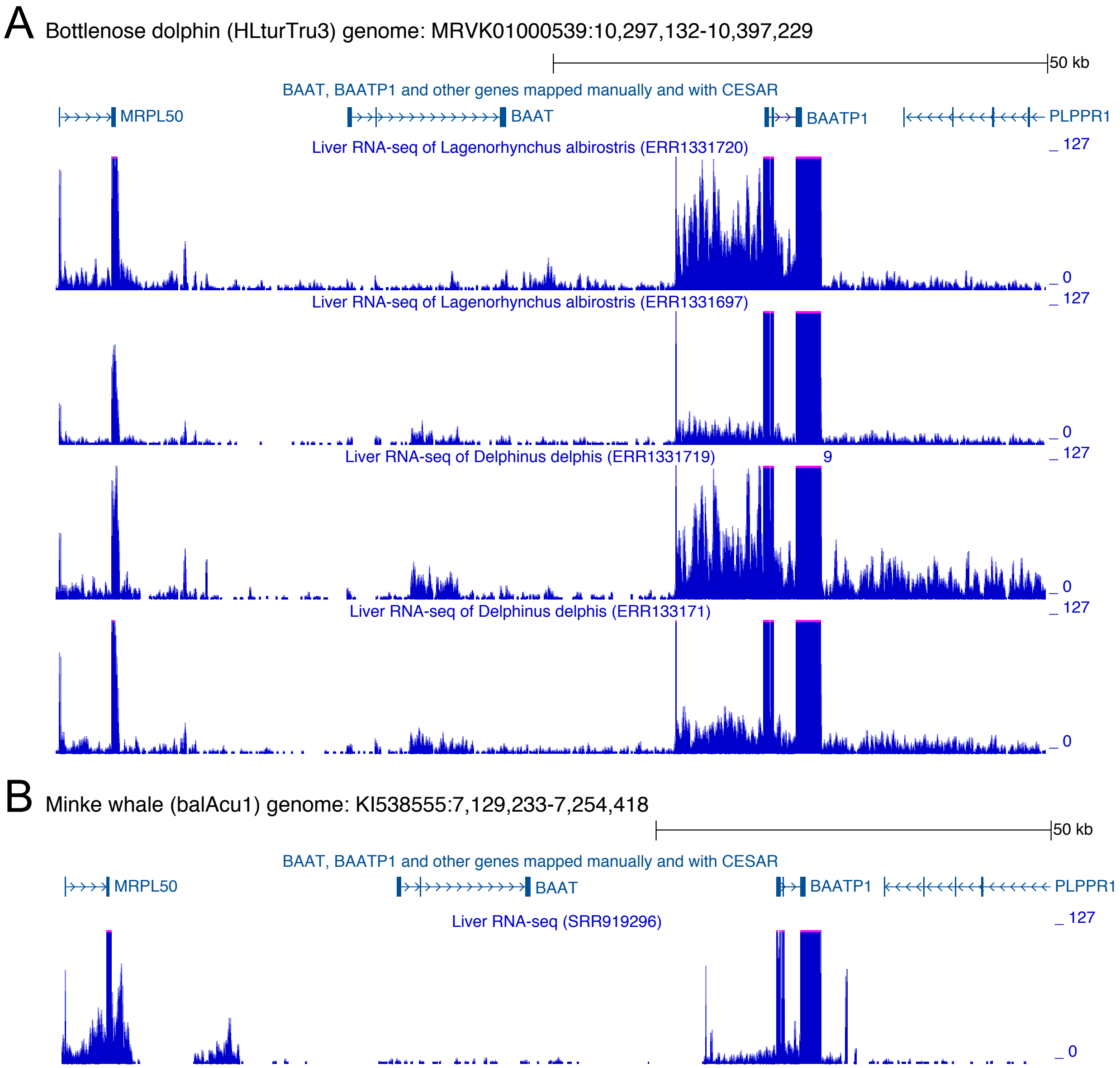

Supplementary Figure 2: Expression of the inactivated *BAAT* and the intact *BAATP1* gene in Delphinoidea and the Minke whale.

(A) UCSC genome browser screenshot showing the *BAAT*/*BAATP1* locus in the bottlenose dolphin genome and RNA-seq data of two related dolphin species (Delphinus delphis and Lagenorhynchus albirostris; (Berthelot, et al. 2018)) that was mapped to bottlenose dolphin genome. In contrast to *BAATP1*, the inactivated *BAAT* gene has no significant exon expression in liver RNA-seq data of two dolphin species.

(B) UCSC genome browser screenshot of the minke whale genome. As for Delphinoidea, only the intact *BAATP1* gene but not the inactivated *BAAT* gene has a significant expression level in liver RNA-seq data.

Mapping of publicly-available RNA-seq data was described in (Hecker, et al. 2019).

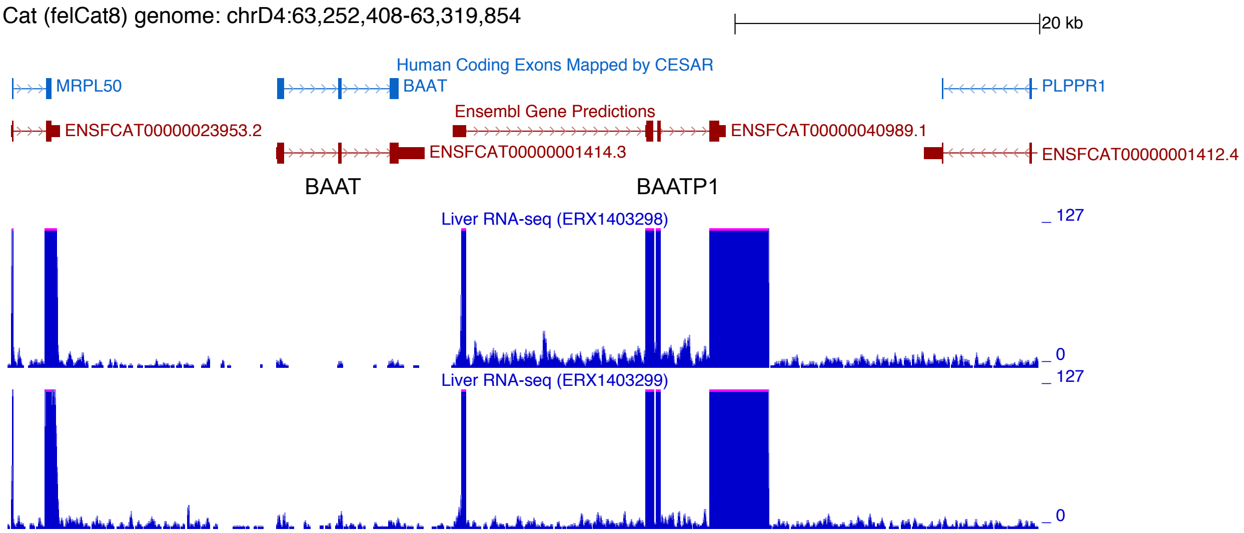

Supplementary Figure 3: Expression of the intact *BAAT* and *BAATP1* gene in cat. UCSC genome browser screenshot shows the *BAAT*/*BAATP1* locus in the cat genome together with liver RNA-seq data (Berthelot, et al. 2018). While both *BAAT* and *BAATP1* have an intact reading frame in cat, only *BAATP1* appears to have a significant expression in liver tissue. Mapping of publicly-available RNA-seq data was described in (Hecker, et al. 2019).

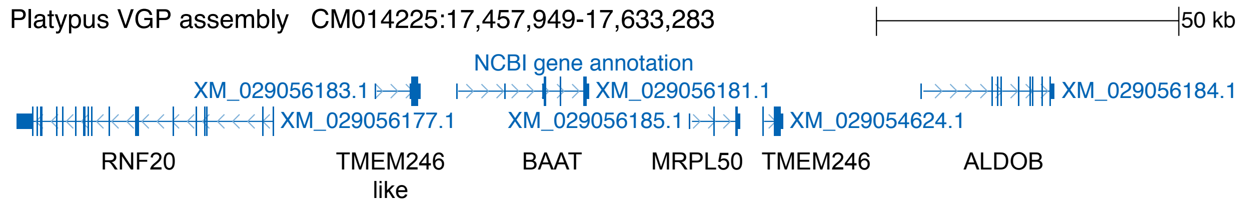

Supplementary Figure 4: *BAAT* locus in the platypus.

UCSC genome browser screenshot showing genes annotated by NCBI (Sayers, et al. 2019) in the newest reference-quality platypus assembly produced by the Vertebrate Genome Project (Zhang, et al. 2019). Annotated gene symbols are shown in black font underneath. Compared to other mammals and non-mammalian species, gene order rearrangements occurred in the platypus that involved the duplication of *TMEM246* and a translocation of *BAAT*. Note that the single *BAAT* gene present in the platypus is flanked by *MRPL50*, just like in other amniotes.

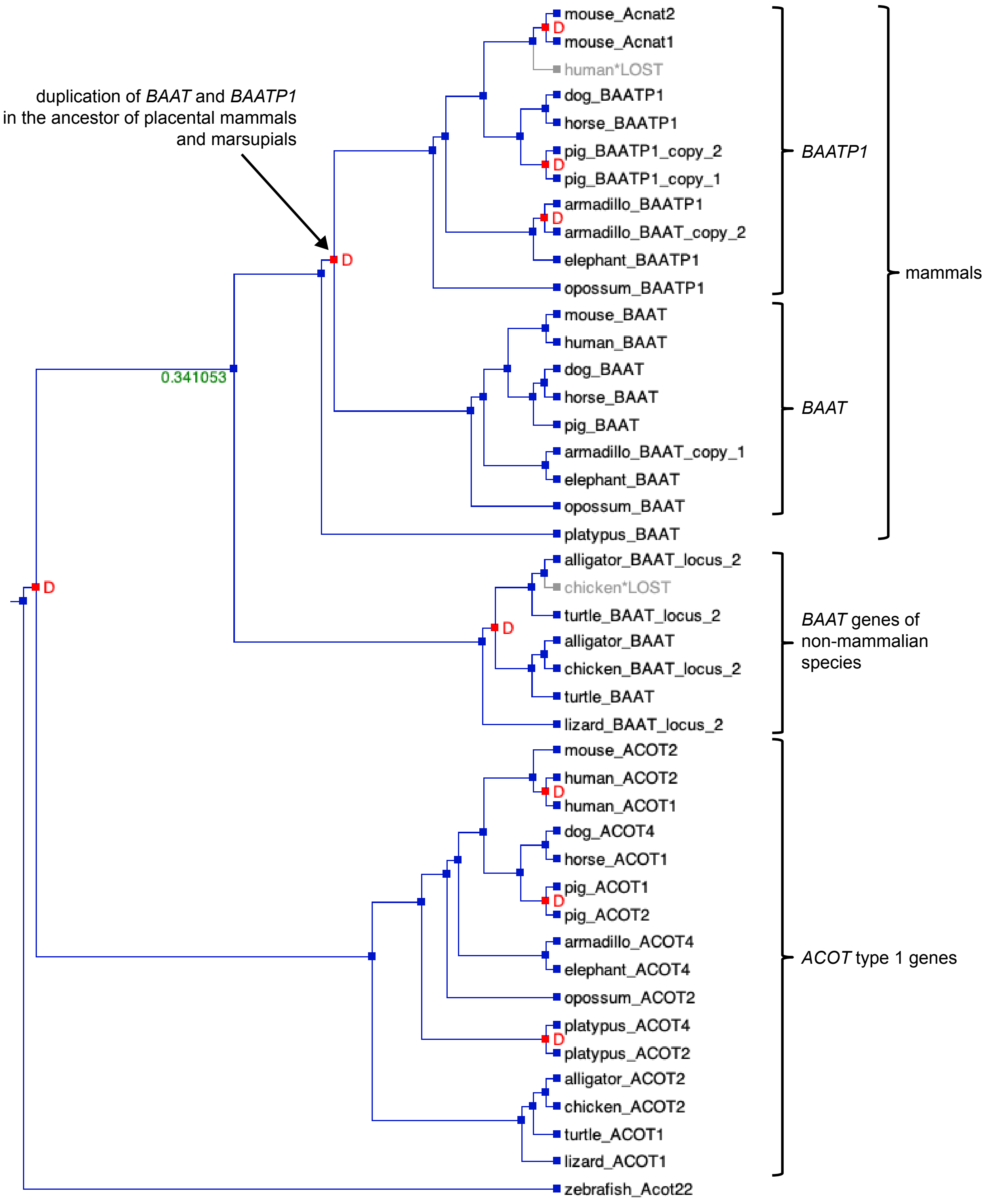

Supplementary Figure 5: *Gene tree-species tree reconciliation of BAAT and ACOT genes.* We used Notung 2.9 (Darby, et al. 2017) to reconcile our gene tree (Figure 3B) with the species tree. Notung supports that (i) *BAAT*/*BAATP1* genes are separated from other *ACOT* family members, (ii) *BAAT* and *BAATP1* genes of placental mammals and marsupials arose by a single

tandem duplication after the split of the monotreme lineage, and (iii) that several placental mammals exhibit additional lineage-specific gene duplications. For the *BAAT* genes in non-mammalian species (chicken, lizard, alligator and turtle) that occur in two separate loci (flanked by *MRPL50* in the first locus and by *ZP1* in the second locus), Notung infers that *BAAT* duplicated in the common ancestor of chicken, alligator and turtle, which was followed by a loss of one copy in chicken. For two reasons, this is likely incorrect. First, the gene tree (Figure 3B) clusters the second-locus *BAAT* genes into a single group, while the first-locus *BAAT* genes of alligator and turtle cluster with the *BAAT* genes of mammals. Second, and more importantly, gene order conservation (Figure 3A) strongly supports that the second-locus *BAAT* genes, which are always flanked by *ZP1*, and the first-locus *BAAT* genes of alligator, turtle and mammals, which are always flanked by *MRPL50*, form two separate groups.

References

Berthelot C, Villar D, Horvath JE, Odom DT, Flicek P 2018. Complexity and conservation of regulatory landscapes underlie evolutionary resilience of mammalian gene expression. Nat Ecol Evol 2: 152-163. doi: 10.1038/s41559-017-0377-2

Darby CA, Stolzer M, Ropp PJ, Barker D, Durand D 2017. Xenolog classification. Bioinformatics 33: 640-649. doi: 10.1093/bioinformatics/btw686

Hecker N, Sharma V, Hiller M 2019. Convergent gene losses illuminate metabolic and physiological changes in herbivores and carnivores. Proc Natl Acad Sci U S A 116: 3036-3041. doi: 10.1073/pnas.1818504116

Sayers EW, et al. 2019. Database resources of the National Center for Biotechnology Information. Nucleic acids research 47: D23-D28. doi: 10.1093/nar/gky1069

Zhang G, et al. 2019. BGI-G10K-VGP platypus male genome, primary haplotype. unpublished.
